# Spatial patterns of preventable perinatal mortality in Salvador, Bahia, Brazil

**DOI:** 10.11606/S1518-8787.2017051007076

**Published:** 2017-08-03

**Authors:** Rita de Cássia de Sousa Nascimento, Maria da Conceição Nascimento Costa, José Uéleres Braga, Márcio Santos da Natividade

**Affiliations:** I Instituto de Saúde Coletiva. Universidade Federal da Bahia. Salvador, BA, Brasil; II Universidade Estadual do Rio de Janeiro. Rio de Janeiro, RJ, Brasil

**Keywords:** Perinatal Death, prevention & control, Perinatal Mortality, Risk Factors, Spatial Analysis

## Abstract

**OBJECTIVE:**

To identify the spatial distribution patterns and areas of higher risk of preventable perinatal mortality in the city of Salvador, State of Bahia, Brazil.

**METHODS:**

We carried out a spatial aggregated study in 2007, considering the weighting areas (census tracts contiguous sets) of Salvador, of which the center and north present low life conditions. Data were obtained from national vital statistics systems and the 2010 Census. Addresses of live births and stillbirths were geocoded by weighting area. The spatial distribution of the perinatal mortality rate was analyzed from thematic maps. Spatial dependence was evaluated by the Global and Local Geary’s and Moran’s Indexes.

**RESULTS:**

Crude and smoothed perinatal mortality rates were high in areas situated to the north, west, and in center of Salvador. The smoothed rates in weighting areas ranged from 4.9/1,000 to 22.3/1,000 births. Of all perinatal deaths, 92.1% could have been prevented. We identified spatial dependence for preventable perinatal mortality for care in pregnancy, with neighboring areas with high risk in the north of the city.

**CONCLUSIONS:**

The preventability potential of perinatal mortality was high in Salvador, in 2007. The spatial distribution pattern with higher rates in disadvantaged areas of the city suggests the existence of social inequalities in health. The characteristics of the process of urban development of Salvador, which has inadequate prenatal care, possibly influenced the magnitude and spatial distribution pattern of this mortality.

## INTRODUCTION

Perinatal mortality has gradually emerged as an important public health problem in developing countries, mainly because of the decline of the post-neonatal component in infant mortality. The study of this mortality can reveal valuable information about the quality of care in pregnancy, delivery, and the newborn’s first days of life. Therefore, it is one of the health measures most used to evaluate quality and access to obstetric and neonatal care and is an indicator of living conditions, since access to quality health care reflects the socioeconomic level of different social groups^1^.

The latest available estimates released by the World Health Organization (WHO) regarding the worldwide mortality of children under five years old show that, in 2009, 41% of these deaths were of children under one year old, of which 75% occurred in the first week of life and 25% in the first day of life. Almost all these deaths (99%) occurred in low- and middle-income countries, being two-thirds of the total in Africa and Southeast Asia, where progress in reducing neonatal and fetal mortality has been slow. As of 2009, in North America and Europe, fetal mortality – the main component of perinatal mortality – remained at around 5/1,000 births and it was lower (2/1,000) in countries such as Finland, Denmark, and Norway. In the Latin America and Caribbean countries, it ranged from 5/1,000 to 25/1,000, while in India, Pakistan, and Nigeria it was 66/1,000, 47/1,000, and 42/1,000, respectively. Despite the disturbing statistics on infant survival, reduction in fetal deaths are not part of the Millennium Development Goals^2^.

In Brazil, government initiatives implemented in the last 20 years, such as the Infant Mortality Reduction Program, Family Health Strategy, and others^3^, have promoted advances throughout the country in the health of children under five years old, particularly in the decrease of infant mortality, which already presents a declining trend^4^. Since the 1990s, when most of infant deaths under one year of age shifted to the neonatal period^a^, this component of infant mortality and perinatal mortality became one of the major challenges in maternal and infant health for the Brazilian health authorities, as well as for health professionals involved in perinatal care. As of late 1990s, early neonatal mortality contributed for the slow decline or stabilization of infant mortality, at relatively high levels, despite the underreporting of information in developing countries, where more than half of causes of perinatal deaths could be prevented by specific actions of health services.

In 2010, the perinatal mortality in Brazil was estimated at 18.1/1,000, being higher in the North and Northeast regions with rates of 19.5/1,000 and 22.1/1,000, respectively, while the South and Southeast had rates of 14/1,000 16/1000, respectively^b^. On the other hand, in countries such as the United States and Canada^5^, the risk of perinatal death is approximately two to three times lower than in Brazil^2^. In Bahia, the largest state in Northeastern Brazil, perinatal mortality has followed the trend of stabilization^b^. In this state, however, no spatial analysis studies have been found in indexed databases about this mortality over the past 15 years. According to the State Committee for Prevention of Infant and Fetal Mortality of 2006, 59.4% of deaths in children under one year old were of infants younger than seven days old, and 84% of them were due to perinatal causes, indicating its high magnitude^c^.

Data of the Health Department shows that the perinatal mortality in Salvador, the capital of the State of Bahia, was 26.1/1,000 in 2007^d^. Two years later, the Committee for Prevention of Infant and Fetal Mortality was created in this city, an important strategy to implement the surveillance of infant death^6,7^. Despite this initiative, perinatal mortality was still high in 2010 (25.8/1,000 in Bahia and 24.5/1,000 in Salvador), highlighting the need for greater surveillance and studies investigating the causes and risk areas, so that such information can support plans of action aimed at its control^6,7^. These data show that such indicators are also useful for analyzing geographical and temporal variations of early neonatal and fetal mortality^2,e^with the identification of trends, determinants, and inequalities.

Studies related to the relation between geographical space, social environment, and health care can contribute to understand causality, especially the importance of life experiences, as the geographical and social space is where various processes involved in the living conditions and health of a population occur and are expressed^8^. Thus, this study aimed to identify spatial distribution patterns of perinatal mortality and areas with higher risk of occurrence of preventable perinatal deaths in Salvador, State of Bahia, Brazil.

## METHODS

A spatial aggregate study of perinatal mortality was conducted in 2007, in Salvador, State of Bahia, Brazil. According to the 2010 Demographic Census data, this city has 693,292 km^2^, 2,675,656 inhabitants, and population density of 3,859.35 persons/km^2^. The analysis units included the 63 weighting areas (WA) of the city, which correspond to 3,538 census tracts. According to the Brazilian Institute of Geography and Statistics (IBGE), WA is a contiguous set of neighboring census tracts, seeking homogeneity, and consisting of at least 400 occupied private households of the sample that answered the questionnaire of the 2010 Demographic Census. In Salvador, areas located at the South, such as those on the Atlantic seafront, show better socioeconomic conditions, while those areas located in Center and North present precarious living conditions. However, it is worth noting that in many areas of the city, the social profile is less clear, since there is great social diversity^f^, expressing the existence of heterogeneity of the social-geographical space of the city.

The study population consisted of live births and stillbirths (between the 22nd week of pregnancy or birthweight ≥ 500 g and the sixth day of life)^9^ of mothers living in Salvador during 2007. Data on births were obtained from the Live Birth Information System (Sistema de Informação sobre Nascidos Vivos – SINASC), while data related to perinatal deaths were acquired from the Mortality Information System (Sistema de Informação sobre Mortalidade – SIM), both provided on magnetic media (CD) by the Municipal Health Department of Salvador. To calculate the perinatal mortality rate (PMR), the total of perinatal deaths in 2007 was used, divided by total sum of live births and stillbirths multiplied by 1,000. In that year, the SIM coverage was over 80% and the SINASC was over 90%, which justified the adoption of that year (2007) to carry out this study^d^.

The geocoding of residential addresses of live births and stillbirths, by WA, was conducted using the Localiza software (Instituto de Saúde Coletiva of the Universidade Federal da Bahia/ISC/UFBA) and Google Earth. The WA 33 was excluded, consisting of seven census tracts, which correspond to islands not offering proximity to the mainland. For analysis, the Arc-View Map GIS software was used.

To minimize random fluctuations from small numbers, PMR (based on total and group of avoidable causes), via WA, was transformed by the Freeman-Tukey smoothing method (square root) in the Stata software (10.0, 2007), and the neighborhood matrix was created. The analysis of regression residuals to identify spatial dependence considered the overall measures of autocorrelation, primarily Global Moran’s Index (values from -1 to +1) and Geary’s Index (values from 0 to 2), using the Stata software (10.0, 2007)^10^. The identification of risk areas (spatial clusters with statistical significance) was performed by Local Moran’s Index/LISA (Local Indicator of Spatial Association) test (p = 0.05), using the OpenGeoDa 1.2.0 version 10. Analysis of spatial distribution patterns in PMR, the total, and by group of preventable causes were carried out from created thematic maps, considering the values of the respective rates by WA, using the ArcView GIS software version 10.

Causes of perinatal deaths were classified using the Brazilian List of Preventable Causes of Death by Interventions of the Brazilian Unified Health System (SUS)^11^, which classifies deaths of children under five years old into preventable causes, ill-defined causes of death, and other causes (not clearly preventable). The preventable causes that were the object of this study are classified as: reducible by preventive actions/vaccines; preventable by appropriate actions of diagnosis and treatment; reducible by adequate health promotion actions; and reducible by sufficient attention to women during pregnancy and delivery, as well as to the fetus and newborn.

The Research Ethics Committee of the Instituto de Saúde Coletiva, Universidade Federal da Bahia, approved this study (Process 182679-12/CEP-ISC).

## RESULTS

In 2007, 36,737 children were born to mothers living in Salvador, and 959 (2.6%) perinatal deaths occurred, of which 431 (44.9%) were in early neonatal period and 528 (55.1%) in the late fetal period. The crude rates of perinatal mortality, early neonatal mortality, and stillbirths were 25.8/1,000, 11.6/1,000, and 14.2/1,000, respectively.

The addresses of 33,174 live births and 831 perinatal deaths were georeferenced. More than half (59%) of these perinatal deaths occurred before delivery and were spread throughout the territory of the city, although a greater proportion was present in the West, South, Center, and North. Approximately 92% of the perinatal deaths were preventable, of which 48.3% could have been prevented by delivery care and 33.5% by adequate care during pregnancy, childbirth, and for newborns (Table). Most of these corresponded to maternal conditions affecting the fetus or newborn (P00, P04), maternal complications during pregnancy that affect the fetus (P01), and disorders related to short gestation and low birthweight, not elsewhere classified (P07). Among the preventable causes by adequate care during delivery, intrauterine hypoxia and birth asphyxia (P20, P21) presented the highest proportion (31.5%) and risk of 11/1,000. The preventable perinatal deaths by appropriate care to the fetus and newborn (18.2%) were mainly due to specific perinatal infections (P35 to P39), which presented the highest magnitude in the South of the city. Congenital anomalies and ill-defined causes accounted for 6.3% and 1.5% of perinatal death causes, respectively.

**Table:** Perinatal deaths: Number and proportion of deaths (%) and perinatal mortality rate (PMR/1,000 births) according to categories of preventability. Salvador, State of Bahia, Brazil, 2007. (n = 831)

Categories of preventability*	n	%	PMR/1,000
By care during pregnancy	256	33.5	7.6
By delivery care	369	48.3	11.0
By care to fetus and newborn	139	18.2	4.1

Total preventable	764	91.9	22.7

Source: SIM; SINASC

Notes: Data for births and deaths whose addresses were geocoded.

Perinatal deaths by congenital anomalies = 54; Perinatal deaths by ill-defined causes = 13.

* According to the List of Preventable Causes of Deaths by Interventions of the Brazilian Unified Health System^11^.

**Figure 1:**
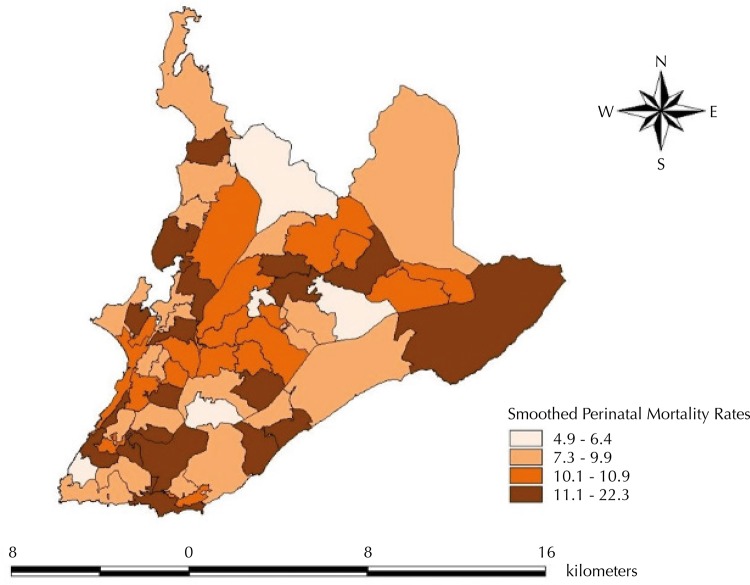
Spatial distribution of smoothed perinatal mortality rates, according to weighting area (WA*). Salvador, State of Bahia, Brazil, 2007.

The spatial distribution of perinatal mortality showed higher crude rates in WA located in the center and some were concentrated in the North, West, South, and East of the city. The smoothed perinatal mortality rates ranged from 4.9/1,000 in WA 63 to 22.3/1,000 births in WA 27 (Figure 1), and their spatial distribution pattern was similar to that described in crude rates.

* WA is a set of geographically contiguous census tracts (aiming for homogeneity), with at least 400 occupied private households that answered the 2010 Census sample questionnaire.

**Figure 2:**
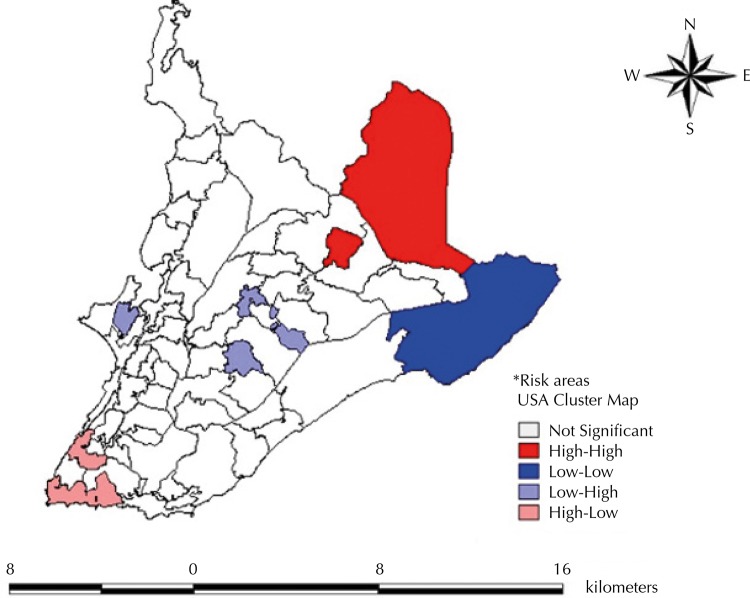
Risk areas* for preventable perinatal mortality by adequate care during pregnancy. Salvador, State of Bahia, Brazil, 2007.

The crude rates of mortality from preventable causes by proper care during pregnancy and delivery were in all areas of the city, while those from preventable causes by proper care to newborn and fetus were more concentrated in WA in the center, northeast, and west of the city. No spatial dependence for spatial distribution of crude and smoothed PMR was detected. Spatial association was only found for perinatal mortality from preventable causes by care during pregnancy (Geary’s Index = 0.915; p = 0.05). The higher rates for these causes were concentrated in areas to the north, center, south, and west. Figure 2 shows that neighboring areas of high risk for perinatal mortality from the causes in this group were located to the north of the city. This study found two WA of positive spatial autocorrelation of high risk (high-high) and one WA of positive spatial autocorrelation of low risk (low-low). Three spatial transition areas (negative spatial autocorrelation), also called intermediate areas, were found; one of them in the South, with high mortality rates and neighboring areas of low risk (high-low), and two WA of low risk and surrounding areas of high risk located to the West and North (low-high). The other areas were not statistically significant by the LISA test.

LISA: Local Indicator of Spatial Association

* According to the Local Moran’s Index (0.05).

## DISCUSSION

The risk of perinatal death in Salvador during 2007 was higher than those recorded in other Northeastern capitals of Brazil^12,g^. As in other large Brazilian cities, fetal mortality was higher than neonatal mortality^a^. Although this finding can be real, we should also consider the possibility of it being due to the disagreement in the definition of live birth and stillbirth, especially when it comes to infants with very low birthweight or gestational age; thus, they have been incorrectly considered as unviable, even when being born alive.

Despite the expansion of perinatal care in Brazil, in the last 20 years^a,g^, the proportion of preventable perinatal deaths in Salvador has been very high. Furthermore, the marked differences in the magnitude of this mortality among WA indicate that this improvement has not produced the advances expected, at least not yet.

Notwithstanding the great heterogeneity of the pattern of occupation of the social-geographical area of Salvador^f^, the highest PMR was located in WA located in the city center, which corresponds to areas where the occupation of the territory began by the low- and middle-class population. They headed towards the railroad suburb, a densely populated area, and the north, which is a disorganized area of more recent occupation and with poor service infrastructure. In these areas, the living conditions are precarious^f^. On the other hand, in the south and east, which are more developed and socially more advantaged, the frequency of perinatal mortality was lower^13^.

Therefore, this spatial distribution pattern reflects the relationship between the living conditions of the different social strata of the population of Salvador and perinatal mortality, expressing social inequalities in health. Moreover, Salvador has one of the lowest coverage of primary health care and this condition may have determined the spatial distribution pattern of preventable perinatal mortality by pregnancy care. This study indicated areas of spatial dependence and transition for the risk of perinatal mortality in the North of the city, where the health care structure is still precarious^d^, while in the southern region, the trend of areas at risk indicated improvement of the inequality between north and south.

Salvador has less than 0.1 obstetric beds per 1,000 users of the SUS^h^, which is not even close to the recommended number for at-risk pregnancies. Besides the lack of obstetric beds, nearly 60% of them are concentrated in the south, and the others are in the center, north, and west^h^, which are areas where PMR is higher and the number of basic health units is smaller. Such factors can also have influenced the magnitude of perinatal mortality. This situation suggests the existence of inequality in access to obstetric and neonatal services, penalizing more remote and less structured areas of the city.

The WHO data showed that 9% of the causes of perinatal death in infants were related to asphyxia and tended to decrease with the level of the country’s development. In this study, the proportion of perinatal deaths from intrauterine hypoxia or birth asphyxia was three times higher and similar to the findings in the national literature^14^. Although potentially avoidable by proper care during the pregnancy, childbirth, and newborn period, these are the most common causes of brain injury that can result in mental retardation, seizures, and cerebral palsy^14^, which therefore entail high costs and can contribute to increase perinatal and infant mortality.

The spatial pattern of perinatal mortality found in this study, in which the poorest areas presented higher perinatal mortality rates, possibly, reflects the relationship between the living conditions of the different social strata of the population of Salvador, expressing social inequalities in health. The characteristics of the process of urban development of this city may have contributed to outline this spatial pattern distribution of perinatal mortality and to determine the high magnitude of their rates.

Some issues need to be considered when interpreting the results of this study, especially those about data quality, such as the underreporting and incorrect or incomplete filling of some of the Death Certificate fields, and errors in the classification of death (fetal or not fetal). In addition, since this is an ecological study aggregated by areas, it is subject to the effects of scale or aggregation of areas, as well as difficulties in defining the geographical boundaries of each area, which can affect the results. Thus, there may have been some heterogeneity intra WA, although certain homogeneity was sought in their definition. In addition, there is the possibility that the results of this study were affected by statistical bias because of the use of smoothed rates. While the stratification by demographic or spatial criteria could be one of the strategies set out to overcome the instability of the rates produced by a small number of observations found in small areas, we chose to smooth the rates and not aggregate the WA. We consider that, for the surveillance, it would be important to estimate perinatal mortality rates in each area in order to allow an indication of specific health actions.

However, despite the limitations, our findings leave no doubt about the need to offer a differentiated prenatal and childbirth care and to stimulate the development of appropriate supervision in areas of higher risk of perinatal death in Salvador. Achieving these purposes requires constant surveillance in areas of high risk, adequate access to quality care provided by qualified professionals, and implementation of public policies aimed at social justice, since these actions can both minimize the adverse effects of disadvantaged social positions in the social space and ensure unquestionable benefits for child survival.

## Footnotes

a Ministério da Saúde (BR), Secretaria de Ciência, Tecnologia e Insumos Estratégicos, Departamento de Ciência e Tecnologia. Síntese de evidências para políticas de saúde: mortalidade perinatal: Brasília (DF); 2012 [cited 2017 Mar 31]. (Série B. Textos Básicos de Saúde). Available from: http://bvsms.saude.gov.br/bvs/publicacoes/sintese_evidencias_mortalidade_perinatal.pdf

b Ministério da Saúde (BR), Rede Interagencial de Informações para a Saúde - RIPSA. Indicadores e dados básicos – Brasil -2007 - IDB-2007. Brasília (DF): RIPSA; 2008 [cited 2013 Nov 16]. Available from: http://tabnet.datasus.gov.br/cgi/idb2007/matriz.htm.

c Ministério da Saúde (BR), Secretaria de Vigilância em Saúde, Secretaria de Atenção à Saúde, Manual de vigilância do óbito infantil e fetal e do Comitê de Prevenção do Óbito Infantil e Fetal. 2. ed. Brasília (DF); 2009 [cited 2017 Mar 31]. (Série A. Normas e Manuais Técnicos). Available from: http://bvsms.saude.gov.br/bvs/publicacoes/manual_obito_infantil_fetal_2ed.pdf

d Prefeitura Municipal de Salvador, Secretaria Municipal de Saúde. Plano Municipal de Saúde 2010-2013. Salvador; 2010 [cited 2017 Mar 31]. Available from: http://www.saude.salvador.ba.gov.br/arquivos/astec/PMS_final.pdf

e Bahia. Secretaria Estadual de Saúde, Superintendência de Vigilância da Saúde, Diretoria de Informação da Saúde. Base de dados estadual [CD-ROOM]. Salvador; 2008.

f Carvalho IMM, Pereira GC. As cidades de Salvador. In: Carvalho IMM, Pereira GC, organizadores. Como anda Salvador e sua Região Metropolitana. 2. ed. Salvador: EDUFBA; 2008 [cited 2017 Mar 31]. Available from: http://static.scielo.org/scielobooks/36d/pdf/carvalho-9788523209094.pdf

g Silva AVS. Tendências e diferenciais na saúde perinatal no município de Fortaleza, Ceará: 1995 e 2005 [these]. São Paulo: Faculdade de Saúde Pública da Universidade de São Paulo; 2010.

h Ministério da Saúde (BR). Cadastro Nacional de Estabelecimentos de Saúde - CNES. Brasília (DF); 2004 [cited 2014 Jan 12]. Available from: www.cnes.datasus.gov.br
